# Engineering dielectric properties and charge transport in PANI/CuO nanocomposites via microstructural control

**DOI:** 10.1038/s41598-025-30835-3

**Published:** 2025-12-15

**Authors:** Noura M. Saleh, Abdelhamid A. Sakr, E. M. El-Maghraby, Saeid M. Elkatlawy

**Affiliations:** 1 Department of Physics, Faculty of Science, Damanhour University, Damanhour, 22111 Egypt; 2 Department of Physics, Faculty of Science, University of Sadat City (USC), El-Sadat City, Egypt

**Keywords:** Polyaniline nanocomposites, Dielectric properties, Correlated barrier hopping (CBH), Copper oxide (CuO), Electrical conductivity, Materials science, Nanoscience and technology, Physics

## Abstract

This study systematically investigates the structure-property relationships in polyaniline/copper oxide (PANI/CuO) nanocomposites, with a specific focus on how controlled CuO incorporation (0.5 to 1.25 mol%) tunes their microstructural, dielectric, and charge transport characteristics. The key innovation of this work lies in establishing a direct correlation between CuO-induced lattice expansion and the evolution of charge transport mechanisms, revealing a tunable microstructural-dielectric coupling. X-ray diffraction confirmed successful composite formation, revealing a significant lattice expansion and an optimized microstructure with increased crystallite size and reduced micro-strain. Dielectric spectroscopy demonstrated a remarkable enhancement in the dielectric constant and revealed a distinct interfacial polarization peak. The analysis of AC conductivity identified Overlapping Large-Polaron Tunneling as the dominant charge transport mechanism, a finding further supported by the calculated trends in hopping distance. Complex impedance analysis confirmed non-Debye relaxation behavior and visualized the critical role of interfacial effects, which transition from blocking to conductive with increasing temperature. The PANI/CuO-1 mol composite emerged as the optimal candidate, achieving an ideal balance between high charge storage and efficient transport. This work not only advances the fundamental understanding of charge dynamics in hybrid systems but also underscores the potential of these tailored nanocomposites for high-performance capacitive and optoelectronic applications.

## Introduction

The manipulation of trapped electric charges at interfaces in conductive polymer nanocomposites enables a diverse array of applications. These applications span diverse sectors, including industrial technology, biotechnology, and medicine, impacting all phases from fundamental research to commercial production. Key dielectric parameters, namely the permittivity (ε′) and dissipation factor (tan δ), are critical for electronic device design. Furthermore, these properties serve as powerful diagnostic tools, providing profound insight into the material’s chemical and physical state through their variation with temperature and frequency. The fundamental electrical response of a polymer is governed by its intrinsic charge distribution and the thermal agitation of its polar constituents. Consequently, analyzing dielectric relaxation behavior offers a powerful methodology for elucidating the nature of molecular motions and their dependence on chemical composition, molecular architecture, and morphology^1–3^. Composite materials integrating transition metal oxides like copper oxide (CuO) with conductive polymers such as polyaniline (PANI) have gathered significant interest. These composites advantage synergistic effects to create materials with enhanced mechanical, thermal, and optoelectronic properties^4–6^. Among conductive polymers, PANI is particularly attractive due to its straightforward synthesis, high electrical conductivity, and environmental stability^7–9^. Similarly, CuO nanoparticles are promising fillers because of their high electrical conductivity, narrow band gap, and pronounced redox activity^5,6^. The combination of PANI and metal oxides has proven effective for applications ranging from gas sensing and catalysis to energy storage and optoelectronics. Despite this promise, a critical challenge persists in optimizing the electronic and charge transport properties of PANI/CuO nanocomposites. While the composite’s potential is widely recognized, its functional performance in devices like supercapacitors and sensors is highly dependent on fundamental characteristics such as dielectric behavior and charge dynamics^10–12^. Furthermore, these properties are not intrinsic but are profoundly influenced by the synthesis method, which governs the composite’s morphology, crystallinity, and interfacial structure.

A comprehensive understanding of how synthesis-controlled structure dictates the structure-property relationship of composite materials^13–15^.

Referring to the dielectric and charge transport properties in PANI/CuO nanocomposites. Many studies report on applications, but fewer provide a deep, multi-faceted characterization that directly links the synthesis process to the atomic structure, morphology, and ultimately, the electronic performance.

A prime example is their use in gas sensing; upon exposure to specific analytes, the composite’s conductivity decreases significantly due to a marked increase in electrical resistance, providing a detectable signal for sensor operation^16–18^. The applicability of PANI/CuO nanocomposites extends to diverse fields. Their utility in chemical catalysis is driven by the intrinsic catalytic properties of copper oxide, which, as a transition element, exhibits multiple oxidation states and magnetic behavior that facilitate redox reactions^19^. Furthermore, these nanocomposites are effective in protective and conductive coatings, significantly enhancing adhesion and surface durability^20^.

Optoelectronic applications represent another major frontier. In hybrid photovoltaic cells, the nanocomposite functions as a bulk heterojunction: the conjugated polymer backbone of PANI (acting as an n-type donor) absorbs light and transports holes, while the p-type semiconductor CuO acts as an electron acceptor, facilitating charge separation^21^. This tunable optoelectronic behavior also makes PANI-based composites excellent candidates for optical chemical sensors, as their electrical conductivity and optical activity are highly responsive to analyte interactions^10–12,22–26^.

While the individual components of PANI and CuO are well-studied, a fundamental understanding of how CuO inclusion directly influences the atomic structure of PANI and, in turn, governs its charge transport mechanisms remains a critical knowledge gap. To address this, the present work establishes a novel correlation between CuO-induced lattice expansion and the evolution of charge transport in PANI/CuO systems. We demonstrate that microstructural dielectric properties can be precisely tuned through controlled filler incorporation. This insight is achieved by synthesizing a series of PANI/CuO nanocomposites via a hydrothermal method, chosen for its precise control over crystallinity and morphology, and employing a targeted characterization strategy. The atomic structure is probed with X-ray diffraction (XRD), the morphology with scanning electron microscopy (SEM), and the charge dynamics through detailed frequency- and temperature-dependent dielectric spectroscopy. Ultimately, this study provides a new correlation between lattice expansion and charge transport mechanisms in PANI/CuO systems, revealing tunable microstructural dielectric coupling via controlled CuO incorporation.

## Materials and methods

### Materials and synthesis

All chemicals were of laboratory analytical grade and used as received. The synthesis of pristine polyaniline (PANI) was carried out via a hydrothermal method. In a typical procedure, aniline hydrochloride (AnHCl, C_6_H_8_ClN, Oxford Laboratory, 99%) was dissolved in 20 mL of distilled water within an ice bath. In a separate container, an equimolar amount (0.25 mol) of ammonium persulfate (APS, (NH_4_)_2_S_2_O_8_, LOBA CHEMIE, 99%) was dissolved in 20 mL of distilled water. The APS solution was then added dropwise to the AnHCl solution under constant stirring for 20 min. The resulting mixture was transferred to a 100 mL Teflon-lined stainless-steel autoclave and subjected to a hydrothermal reaction at 180 °C for 3 h. After natural cooling to room temperature, the resulting precipitate was collected and repeatedly washed with distilled water and ethanol until the filtrate reached a neutral pH (~ 7). The final product was dried in an oven at 180 °C to obtain pristine PANI.

The PANI/CuO nanocomposites were synthesized via an in-situ hydrothermal method. The synthesis began with the preparation of a polyaniline (PANI) precursor. An equimolar amount (0.25 mol) of aniline hydrochloride (AnHCl) was dissolved in 20 mL of distilled water under cooling in an ice bath. Separately, 0.25 mol of ammonium persulfate (APS) was dissolved in 20 mL of distilled water. The APS solution was then added dropwise to the AnHCl solution under constant stirring for 20 min to initiate polymerization.

Concurrently, a copper oxide (CuO) precursor was prepared by a chemical co-precipitation route. A specified amount of copper sulfate pentahydrate (CuSO_4_·5H_2_O) was dissolved in 20 mL of distilled water. This solution was transferred to a beaker and stirred at 30 rpm on a magnetic stirrer with a hot plate preheated to 75 °C. Subsequently, an aqueous solution of sodium hydroxide (NaOH, 6 mol in 10 mL distilled water) was added to the copper sulfate solution. The addition of NaOH induced a color change from blue to black, indicating the formation of copper oxide nanoparticles in-situ.

The CuO precursor mixture was then combined with the PANI precursor and stirred for an additional 15 min to ensure homogeneity. The final reaction mixture was transferred to a 100 mL Teflon-lined stainless-steel autoclave and subjected to hydrothermal treatment at 180 °C for 3 h. After natural cooling to room temperature, the resulting precipitate was collected and washed repeatedly with distilled water and ethanol until the filtrate reached a neutral pH (~ 7). The final product was dried in an oven at 180 °C to obtain the PANI/CuO nanocomposite powder.

To investigate the effect of filler concentration, a series of composites were synthesized by varying the amount of CuSO_4_·5H_2_O at 0.5, 0.75, 1.0, and 1.25 mol, while keeping the concentration of the PANI matrix constant.

### Pellet preparation and electrode configuration

The synthesized PANI/CuO nanocomposite powders were processed into pellets for electrical characterization. A precise amount of 0.1 g of each powder sample was uniaxially pressed into a pellet using a hydraulic press. The pressing was carried out under a load of 5 tons with a dwell time of 5 min to ensure mechanical stability and uniformity.

For the electrical measurements, ohmic contacts were established by coating the two circular faces of each pellet with a high-purity silver paste. The electrodes were cured at room temperature for 1 h to ensure optimal adhesion and electrical contact. Pelletized samples with a diameter of ~ 10 mm and a thickness of ~ 0.35 mm were prepared.

### Dielectric spectroscopy measurements

The dielectric properties of the pelletized PANI/CuO nanocomposites were characterized using a broadband dielectric impedance analyzer (LCR HiTESTER 3532-50, Hioki, Japan). Measurements were performed over a frequency range of 100 Hz to 5 MHz at room temperature. An excitation voltage of 1.0 V was applied across the silver-pasted electrodes of the pellets. To ensure measurement accuracy and minimize external electromagnetic interference, the samples were measured using a two-terminal shielded fixture.

For temperature-dependent measurements, the sample pellet was housed in a custom-designed, temperature-controlled oven. The temperature was regulated by a programmable PID controller, allowing for stable measurements across a range from 293 K to 373 K with an estimated accuracy of ± 1 K.

### Characterization

The structural characteristics, composition, and physical state of the synthesized materials were analyzed using wide-angle X-ray powder diffraction (XRD). Measurements were performed on an APD 2000 Pro diffractometer (GNR, Italy) utilizing Cu-Kα radiation (λ = 1.5406 Å). Diffraction patterns were acquired over a 2θ range of 5° to 80° with an operating voltage of 35 kV and a current of 25 mA. The analysis of surface morphology by SEM, for the PANI/CuO nanocomposite have been extensively described in a previous publication^9^.

## Results and discussion

### XRD analysis

The XRD analysis, detailed previously^9^, confirmed the successful incorporation of crystalline copper oxide into the PANI matrix. The patterns showed distinct peaks for monoclinic CuO and cubic Cu_2_O, confirming the retention of crystalline structure within the composite and the self-reduction of copper oxide.

Herein we discuss the key microstructural parameters - namely the lattice constant, crystallite size, and micro-strain - derived from XRD analysis.

The experimental lattice constant ($\:{a}_{exp}$) was calculated using the below equation:

1 $$\:{a}_{exp}={d}_{hkl}\sqrt{{h}^{2}+{k}^{2}+{l}^{2}}$$

where d is the interplanar spacing and (ℎ, k, l) are the Miller indices of the corresponding diffraction peak.

The average crystallite size of the CuO nanoparticles was subsequently estimated using Scherrer’s formula^27^.

2 $$\:D\:=\frac{\:\:\:K\lambda\:\:}{\beta\:\:cos\:\theta\:}$$

where *K* is the shape factor (0.89), D is the crystallite size (nm), λ is the X-ray wavelength of Cu Kα radiation, and β is the corrected full width at half maximum (FWHM) of the diffraction peak (in radians).

Furthermore, the micro-strain (ε) within the CuO nanoparticles for the composite sample was calculated using the following relation:

3 $$\:\epsilon\:=\frac{\beta\:}{4\mathrm{tan}\theta\:}\:$$

**Table 1:** Lattice constant $\:{a}_{exp}$, crystallite size, and micro-strain for pure PANI and PANI/CuO-1, 2, 3, 4. Having 1 = 0.5 mol, 2 = 0.75 mol, 3 = 1 mol, 4 = 1.25 mol.

Sample	Phase	2θ (deg)	d-spacing (Å)	Lattice Parameter (Å)	Crystallite Size, D (nm)	Micro-strain, ε (×10^− 3^)
PURE	PANI	20.19 (Halo)	4.394	–	–	–
	PANI	25.57 (Halo)	3.481	–	–	–
1	CuO	35.7	2.513	a = 4.35, b = 3.43, c = 5.13	161	7.03
	Cu_2_O	38.93	2.311	a = 4.00	152	6.86
2	CuO	35.84	2.504	a = 4.34, b = 3.42, c = 5.12	211	5.33
	Cu_2_O	39.04	2.305	a = 3.99	207	5.02
3	CuO	35.63	2.518	a = 4.36, b = 3.44, c = 5.14	298	3.8
	Cu_2_O	38.82	2.318	a = 4.02	163	6.39
4	CuO	35.67	2.515	a = 4.36, b = 3.44, c = 5.14	329	3.44

Table 1 summarizes the key structural parameters derived from XRD analysis. A systematic evolution in the properties of the copper oxide phases is evident with increasing precursor concentration. The crystallite size of the dominant CuO phase increases consistently from 161 nm (PANI/CuO-1) to 329 nm (PANI/CuO-4), while the micro-strain systematically decreases from 7.03 × 10^− 3^ to 3.44 × 10^− 3^. This inverse relationship is a classic materials phenomenon, indicating that higher precursor concentrations promote the growth of larger, more structurally relaxed CuO nanoparticles. These results are in good agreement with published data^12,28^.

Furthermore, the quantitative analysis confirms the co-existence of both CuO and Cu_2_O phases, with Cu_2_O being most prominent in samples 1 and 2. This provides direct evidence for the self-reduction process during synthesis. For the PANI matrix, the d-spacings of its characteristic amorphous halos (~ 4.4 Å and ~ 3.5 Å) remain largely constant, confirming that the fundamental semi-crystalline structure of the polymer is preserved. The controlled microstructural evolution of the oxide nanoparticles, characterized by increased crystallite size and reduced strain, is a fundamental factor governing the dielectric properties discussed in the subsequent section.

### Dielectric measurement

To probe the polarization mechanisms and relaxation processes, dielectric relaxation spectroscopy was employed to study the frequency-dependent (100–5 MHz) and temperature-dependent (293–373 K) dielectric properties of pure PANI and the PANI/CuO nanocomposites.

#### Frequency and temperature dependence of dielectric constant (ε′)

**Fig. 1:**
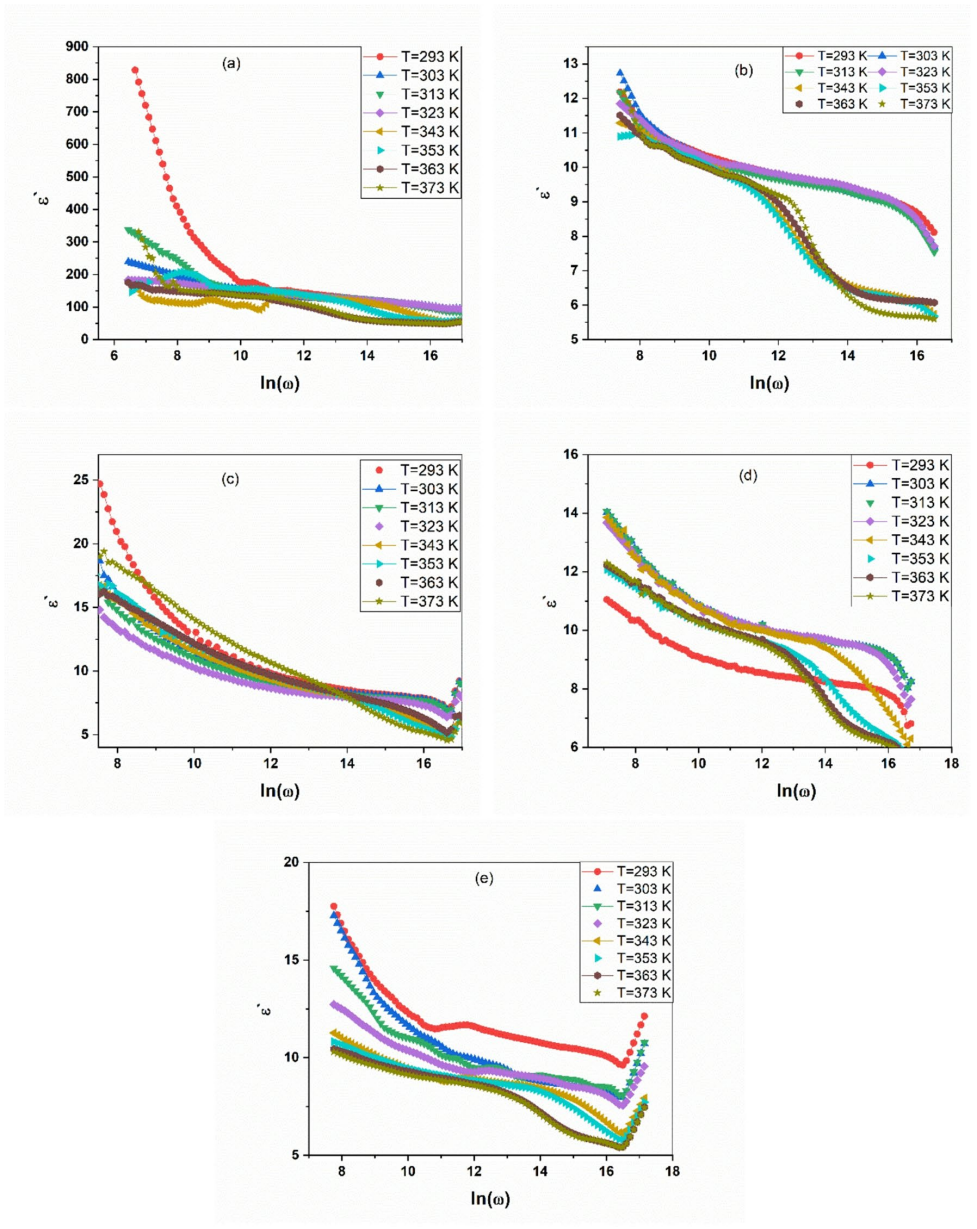
Frequency-dependent dielectric constant (ε′) for pure PANI and the PANI/CuO nanocomposite series, measured across a wide temperature range (293–373 K). (**a**) pure PANI, (**b**) PANI /0.5 mol CuO, (**c**) PANI /0.75 mol CuO, (**d**) PANI/1 mol CuO, and (**e**) PANI /1.25 mol CuO.

The frequency-dependent dielectric constant (ε′) for pure PANI and the PANI/CuO nanocomposite series, measured across a wide temperature range (293–373 K), is presented in Fig. 1a–e. The data reveals a strong correlation between composition, molecular structure, and dielectric response, governed by polarization mechanisms and charge transport dynamics.

All samples exhibit a universal dielectric response, with ε′ decreasing sharply at low frequencies before stabilizing at higher frequencies. This dispersion is characteristic of dielectric relaxation, where slower polarization mechanisms cannot follow the rapid alternation of the electric field. The exceptionally high ε′ values at low frequencies, particularly for the nanocomposites, are suggestive of a Maxwell-Wagner-Sillars (MWS) interfacial polarization mechanism^29–31^. In these heterogeneous systems, charge carriers within the conductive PANI matrix accumulate at the interfaces with the insulating CuO nanoparticles, creating large macroscopic dipoles.

The optimal dielectric performance is observed for the PANI/CuO-0.75 mol and PANI/CuO-1 mol composites. This aligns with the structural data from XRD, which indicated that these compositions achieve an ideal balance: sufficient CuO content to create a high density of interfacial polarization, while maintaining a well-connected PANI network for efficient charge transport. The degradation in performance for the PANI/CuO-1.25 mol sample suggests excessive filler content begins to disrupt the conductive network, hindering charge transport to the interfaces.

Furthermore, a significant thermal activation of ε′ is observed across all compositions. Rising temperature enhances charge carrier mobility and reduces the polymer’s internal viscosity, facilitating both the migration of charges to interfaces and the orientation of dipoles. This strong temperature dependence confirms that the dielectric response is a thermally activated process^32–35^. The convergence of ε’ values at high frequencies for all temperatures indicates a regime where only fast, electronic polarization, insensitive to thermal effects, remains active.

#### Dielectric loss (ε″)

**Fig. 2:**
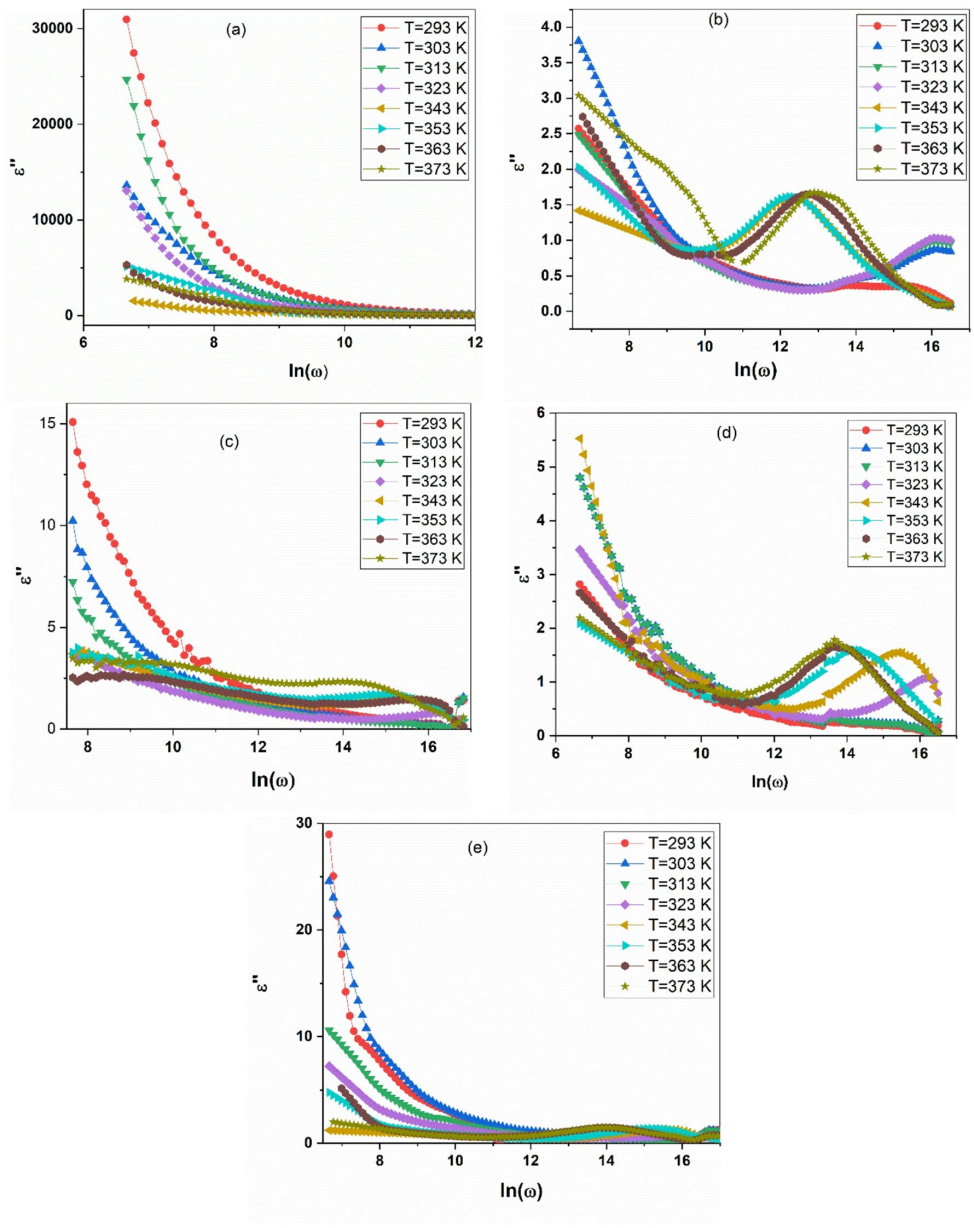
Frequency-dependent dielectric loss (ε″) for pure PANI and the PANI/CuO nanocomposite series, measured across a wide temperature range (293–373 K). (**a**) pure PANI, (**b**) PANI/0.5 mol CuO, (**c**) PANI/0.75 mol CuO, (**d**) PANI/1 mol CuO, and (**e**) PANI/1.25 mol CuO.

The frequency-dependent dielectric loss (ε″) for the PANI/CuO nanocomposites is presented in Fig. 2a–e. The data reveals a strong transition in the dominant loss mechanism, driven by composition and temperature, moving from conduction-dominated behavior to clear interfacial relaxation.

Pure PANI Fig. 2a exhibits an exceptionally high ε″ at low frequencies, which decreases sharply with increasing frequency. This is characteristic of dominant DC conduction losses, where mobile charge carriers (polarons) in the conductive polymer matrix cause significant energy dissipation through long-range hopping^9,36^. The immense magnitude of ε″ (> 10,000) underscores the high intrinsic conductivity of the emeraldine salt form of PANI.

The inclusion of CuO nanoparticles fundamentally alters this behavior. For all composites Fig. 2b–e, the loss curves evolve to display a prominent, broad peak. This is the signature of a dielectric relaxation process, suggests that Maxwell–Wagner–Sillars (MWS) interfacial polarization is likely the dominant mechanism^29,37^. Charge carriers are now trapped and released at the PANI/CuO interfaces, leading to a maximum in energy loss at a specific relaxation frequency (fₘₐₓ).

The systematic evolution of this peak with composition is critical. The relaxation peak is weakest and least defined in PANI/CuO-0.5 mol (b), becomes most prominent and well-defined in PANI/CuO-0.75 mol (c) and PANI/CuO-1 mol (d), and then becomes subdued again in PANI/CuO-1.25 mol (e). This trend mirrors the optimal performance seen in the dielectric constant data, confirming that the 0.75–1 mol% compositions create the ideal density of interfaces for strong interfacial polarization without overly blocking the charge transport needed to activate these interfaces. Furthermore, for all composites, the relaxation peak shifts systematically to higher frequencies with increasing temperature. This confirms relaxation is a thermally activated process. The applied thermal energy assists charge carriers in overcoming the energy barriers at the interfaces, allowing them to relax more quickly. The low-frequency side of the curves remains steep, indicating that DC conduction losses, while suppressed compared to pure PANI, still contribute significantly, especially at elevated temperatures^38,39^.

#### AC conductivity and charge transport mechanism

**Fig. 3:**
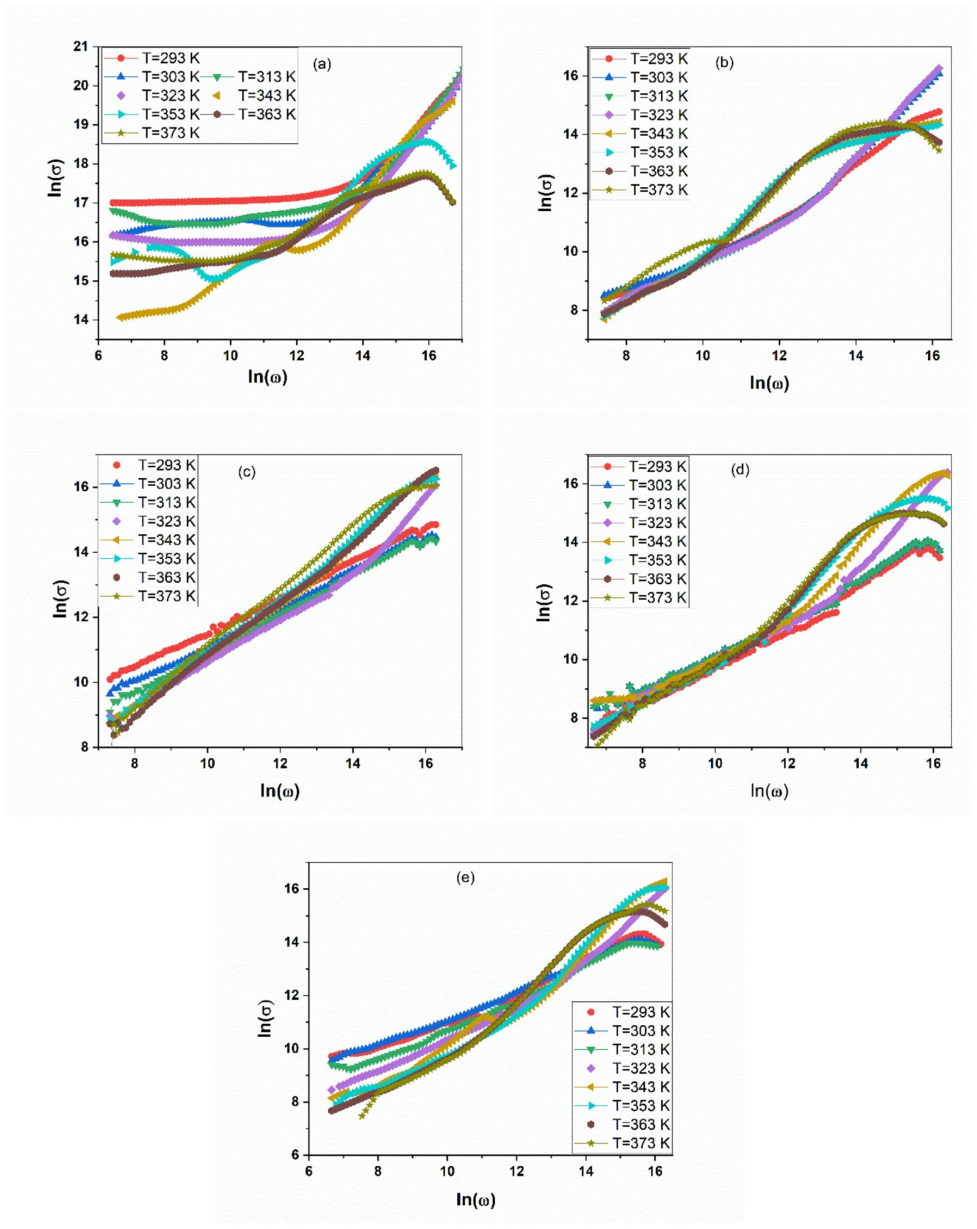
AC conductivity for pure PANI and the PANI/CuO nanocomposite series, measured across a wide temperature range (293–373 K). (**a**) Pure PANI, (**b**) PANI/0.5 mol CuO, (**c**) PANI/0.75 mol CuO, (**d**) PANI/1mol CuO, and (**e**) PANI/1.25 mol CuO.

The AC conductivity$\:{\sigma\:}_{ac}$ spectra for the PANI/CuO nanocomposites are presented in Fig. 3a–e. The data universally exhibits a characteristic pattern: a frequency-independent plateau at lower frequencies, which transitions into a dispersive region where conductivity increases with frequency. This behavior is accurately described by Jonscher’s universal power law^40^, $\:{\sigma\:}_{ac}\left(\omega\:\right)={\sigma\:}_{dc}+A{\omega\:}^{s}$.

A pivotal observation is the systematic enhancement of the DC conductivity plateau $\:{\sigma\:}_{dc}$ with increasing temperature for all samples. This confirms that the fundamental charge transport is a thermally activated process. The $\:{\sigma\:}_{dc}$ values are highest for the PANI/CuO-0.75 mol and PANI/CuO-1 mol composites, directly mirroring the optimal performance observed in both the dielectric constant and loss analyses. This confirms that these compositions achieve the ideal balance, using the structural framework from XRD, where sufficient CuO creates interfaces for polarization without disrupting the continuous conductive pathways necessary for efficient long-range charge transport^41,42^.

The frequency-dependent region provides the microscopic mechanism. The calculated frequency exponent $\:s$ exhibits a clear decrease with increasing temperature (as listed in Table 2). This specific thermal dependence is the definitive signature of the Correlated Barrier Hopping (CBH) model^43,44^.

**Table 2:** Experimental values of the frequency exponent (s) for pure PANI and PANI/CuO nanocomposites at different temperatures.

T (K)	PANI	PANI/CuO-1	PANI/CuO-2	PANI/CuO-3	PANI/CuO-4
293	0.25621	0.77347	0.53975	0.66404	0.53976
303	0.30883	0.85477	0.56635	0.63147	0.51261
313	0.30226	0.90946	0.59883	0.62906	0.56608
323	0.33823	0.91083	0.74827	0.84547	0.75304
343	0.55527	0.85728	0.85773	0.88891	0.8805
353	0.37633	0.84812	0.87017	0.92788	0.92097
363	0.28481	0.86177	0.89345	0.90686	0.91722
373	0.26364	0.77737	0.90119	0.92469	0.9973

In the CBH model, charge carriers (polarons in PANI) hop over a potential barrier between localized sites, where the height of the barrier is correlated with the distance between sites. The decrease in $\:s$ with temperature means that thermal energy assists carriers in overcoming larger barriers, making the conduction less dependent on the assisting electric field frequency. This mechanism dominates over other models like Quantum Mechanical Tunneling, firmly establishing CBH as the core conduction process in this nanocomposite system. That is to say, the AC conductivity analysis completes the structure-property narrative: the optimized microstructure of the PANI/CuO composites facilitates efficient charge transport via a correlated barrier hopping mechanism, leading to superior electrical performance. The analysis of the frequency exponent $\:s$ (Table 2) in conjunction with the AC conductivity spectra confirms the Overlapping Large-Polaron Tunneling (OLPT) model as the dominant conduction mechanism in the PANI/CuO nanocomposites^45–47^.

The consistent increase in the value of $\:s$ with rising temperature provides definitive evidence for this model. This trend is a direct consequence of the conduction process observed in the AC data: as temperature increases, the thermal energy enables polarons to tunnel over larger distances. This expanded tunneling range enhances the interaction between sites, which manifests in the AC spectra as a stronger frequency dependence in the dispersive region, quantified by the increasing exponent $\:s$. This finding completes the charge transport narrative, linking the macroscopic AC conductivity behavior directly to this specific microscopic hopping mechanism^15,48,49^.

#### Complex impedance and electrical relaxation (The Cole-Cole Plots)

**Fig. 4:**
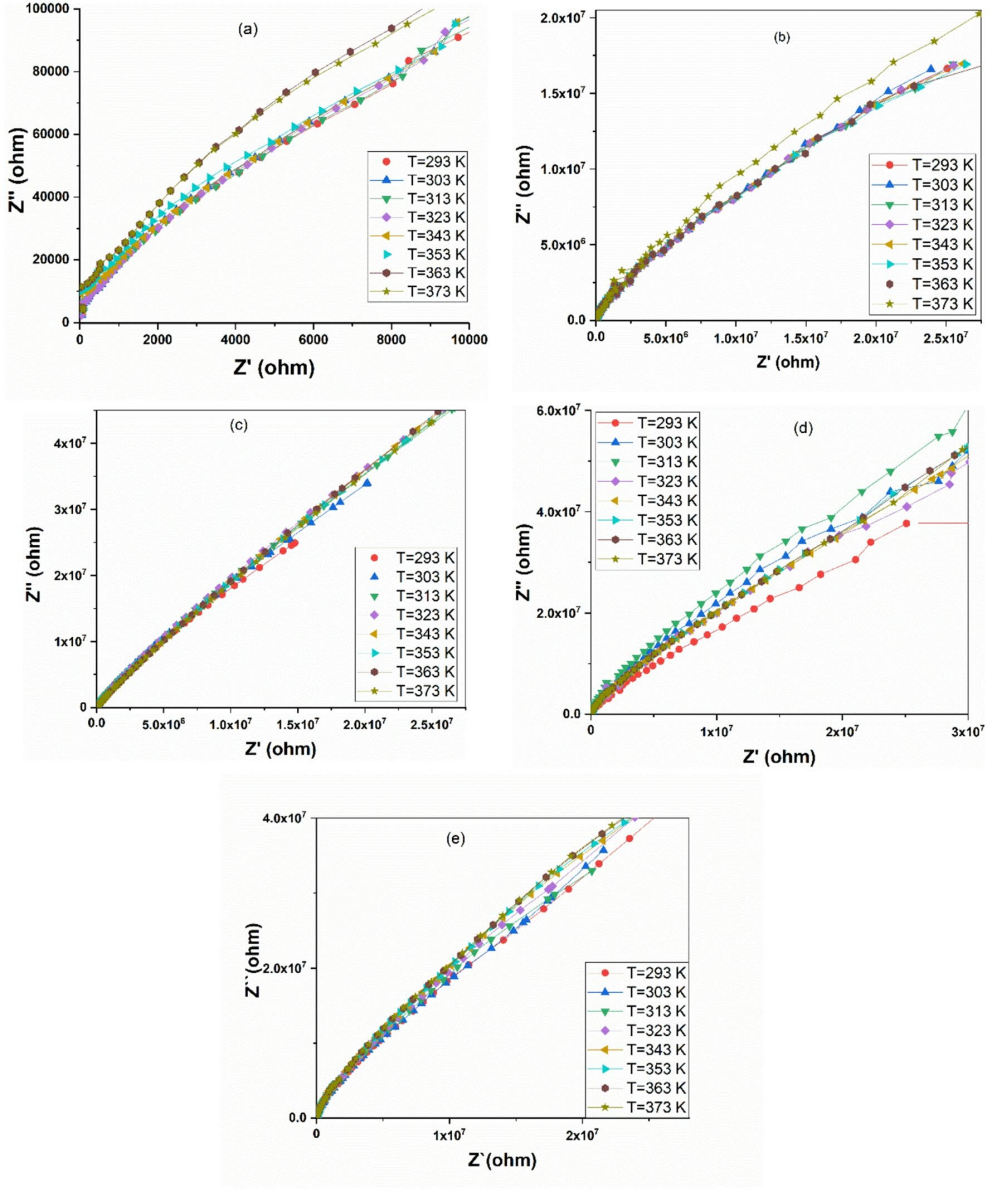
Cole-Cole Plots for pure PANI and the PANI/CuO nanocomposite series, measured across a wide temperature range (293–373 K). (**a**) Pure PANI, (**b**) PANI/0.5 mol CuO, (**c**) PANI/0.75 mol CuO, (**d**) PANI/1mol CuO, and (**e**) PANI/1.25 mol CuO.

The electrical properties of the PANI/CuO composites were further investigated through complex impedance spectroscopy. The Nyquist plots (Cole-Cole arcs) for the composites, presented in Fig. 4a–e, exhibit depressed semicircles, a characteristic feature of non-Debye type relaxation processes^50^. This indicates a distribution of relaxation times within the material, which is typical for heterogeneous systems like conducting polymer composites^28^. Furthermore, a systematic collapse of the semicircular arcs is observed with increasing temperature, suggesting a thermally activated reduction in the overall electrical resistance^51,52^.

The inclusion of CuO nanoparticles fundamentally alters this picture. The composites Fig. 4b–e display impedance spectra that are orders of magnitude larger than pure PANI at low temperatures, evolving from a single arc to spectra suggestive of multiple, overlapping relaxation processes. This is particularly evident in the PANI/CuO-0.5 mol and PANI/CuO-0.75 mol samples Fig. 4b,c, where the data does not form a perfect semicircle.

The most significant finding is the dramatic, thermally activated transformation in the optimal composites. For PANI/CuO-1 mol and PANI/CuO-1.25 mol Fig. 4d,e, the immense semicircles observed at 293 K collapse radically as temperature increases. This indicates a profound reduction in both bulk and interfacial resistance. The near disappearance of the arc at the highest temperatures demonstrates that thermal energy effectively activates charge transport across the PANI/CuO interfaces, transforming them from insulating barriers into conductive pathways. This collapse of the impedance arc provides direct electrical evidence for the superior conductivity and optimized interface structure of these compositions, which was consistently inferred from the dielectric constant, loss, and AC conductivity data. This confirms the critical role of interfaces, demonstrates the thermally activated nature of conduction, and identifies the compositions where the filler content optimally modifies the electrical microstructure to enhance overall performance.

**Fig. 5:**
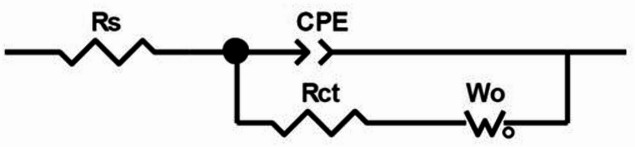
Equivalent circuit fitting of the PANI/CuO interface. R_s_ is the bulk resistance, R_ct_ is charge transfer resistance, CPE is a constant phase element representing the non-ideal double-layer capacitance, W_o_ is the Warburg element, related to ion diffusion or the low-frequency conductive behavior.

The observed non-ideal arcs and their evolution with temperature are consistent with a well-established electrical model for composite materials. A full equivalent circuit fitting of the Nyquist plots is shown in Fig. 5. As reported in the literature for similar PANI-based composites^53^, the impedance behavior can be quantitatively described by a circuit of the type (R_s_–(R_ct_||CPE)–W_o_), where R_s_ represents the bulk resistance, R_ct_ represents the charge transfer resistance at the internal PANI/CuO interfaces, CPE is a constant phase element representing the non-ideal double-layer capacitance, W_o_ is the Warburg element, related to ion diffusion or the low-frequency conductive behavior. The combination (CPE–R_ct_–W_o_) represents the capacitive impedance element of the PANI-CuO interface.

## Conclusions

This work successfully establishes a clear pathway between molecular-level structure and macroscopic electronic function in polyaniline-copper oxide (PANI/CuO) nanocomposites. We have demonstrated that the strategic integration of CuO nanoparticles directly engineers the PANI microstructure, as confirmed by XRD analysis which revealed lattice expansion and optimized crystallite geometry. This structural tailoring is the fundamental driver of the material’s enhanced electrical performance. Engineered microstructure gives rise to two key phenomena: a massive enhancement in charge storage capability and a distinct mechanism for charge transport. The former is driven by interfacial polarization at the newly created PANI/CuO boundaries, leading to a dramatically increased dielectric constant. The latter is governed by a thermally activated overlapping large-polaron tunneling mechanism, as definitively identified through AC conductivity analysis. The radical collapse of impedance observed in Cole-Cole plots at elevated temperatures provides vivid, direct evidence that the interfaces in the optimal composite transform from resistive barriers into highly conductive pathways. The prevalent non-Debye behavior confirms the material’s heterogeneity, while the thermally induced collapse of impedance arcs confirms the critical role of interfaces and identifies the compositions where filler content optimally modifies the electrical microstructure to enhance overall performance. The culmination of this investigation is the identification of PANI/CuO-1 mol as the optimal composition. This material achieves perfect synergy, creating a high density of interfaces for immense polarization without compromising the crystalline order necessary for efficient charge percolation. In summary, this research transforms PANI from a simple conductive polymer into a sophisticated electro-active material. By establishing a precise structure-property relationship, we provide not just a deep understanding of charge dynamics, but also a versatile and powerful platform for developing next-generation capacitors, sensors, and flexible electronic devices. A future detailed study focusing on the temperature-dependent relaxation dynamics, including the extraction of activation energies and scaling behavior, would be highly valuable to fully elucidate the charge transport mechanism.

## Data Availability

The data associated with the work are all involved in the manuscript.
