# Correction: Engineering dielectric properties and charge transport in PANI/CuO nanocomposites via microstructural control

**DOI:** 10.1038/s41598-026-42676-9

**Published:** 2026-03-13

**Authors:** Noura M. Saleh, Abdelhamid A. Sakr, E. M. El-Maghraby, Saeid M. Elkatlawy

**Affiliations:** 1 Department of Physics, Faculty of Science, Damanhour University, Damanhour, 22111 Egypt; 2 Department of Physics, Faculty of Science, University of Sadat City (USC), El-Sadat City, Egypt

Correction to: *Scientific Reports* 10.1038/s41598-025-30835-3, published online 15 December 2025

The original version of this Article contained an error in the caption of Table 2, which was incorrectly given as:

“The values of s as calculated from (CBH) for PNAI and PANI with different loadings of CuO at different temperatures.”

now reads:

“Experimental values of the frequency exponent (s) for pure PANI and PANI/CuO nanocomposites at different temperatures.”

The original Article has been corrected.

